# TNFSF14 (LIGHT) in intestinal inflammation: balancing immune activation and resolution in IBD

**DOI:** 10.3389/fimmu.2025.1657071

**Published:** 2025-09-15

**Authors:** Rabia S. Mousa, Pietro Invernizzi, Joanne L. Jones, Hani S. Mousa

**Affiliations:** ^1^ Department of Medicine and Surgery, University of Pavia, Pavia, Italy; ^2^ Division of Gastroenterology, Centre for Autoimmune Liver Diseases, European Reference Network on Hepatological Diseases (ERN RARE-LIVER), IRCCS Fondazione San Gerardo dei Tintori, Monza, Italy; ^3^ Department of Medicine and Surgery, University of Milano-Bicocca, Monza, Italy; ^4^ Department of Clinical Neurosciences, University of Cambridge, Cambridge, United Kingdom

**Keywords:** LIGHT (TNFSF14), HVEM (TNFRSF14), LTβR (Lymphotoxin-β receptor), DcR3 (TNFRSF6B), BTLA (B and T lymphocyte attenuator), inflammatory bowel disease (IBD), Crohn’s disease, ulcerative colitis

## Abstract

Inflammatory Bowel Disease (IBD), encompassing Crohn’s disease and ulcerative colitis, is an umbrella term used to describe a group of autoimmune conditions characterized by chronic, relapsing inflammation of the gastrointestinal tract. The tumour necrosis factor superfamily member 14 (TNFSF14), also known as LIGHT, is a pleiotropic cytokine with diverse roles in immune regulation. Here, we review the multifaceted involvement of LIGHT in intestinal inflammation, particularly its dual capacity to both promote immune activation and facilitate inflammation resolution in the context of IBD. We explore the molecular mechanisms of LIGHT signalling through its receptors, Herpes Virus Entry Mediator (HVEM) and Lymphotoxin-β Receptor (LTβR), and how these distinct interactions dictate its pro-inflammatory or regulatory functions. Finally, we review the therapeutic potential of targeting this pathway, highlighting the results of recent clinical trials and exploring future strategies aimed at restoring immune homeostasis in patients with IBD.

## 1 Introduction

Inflammatory bowel diseases (IBD), which include Crohn’s disease (CD) and ulcerative colitis (UC), are chronic, relapsing conditions driven by a disruption of intestinal immune homeostasis. The pathogenesis is multifactorial, involving genetic susceptibility (1), environmental triggers, an altered gut microbiota, and a dysregulated immune response that leads to persistent inflammation of the gastrointestinal tract (2–4).

**Figure 1:**
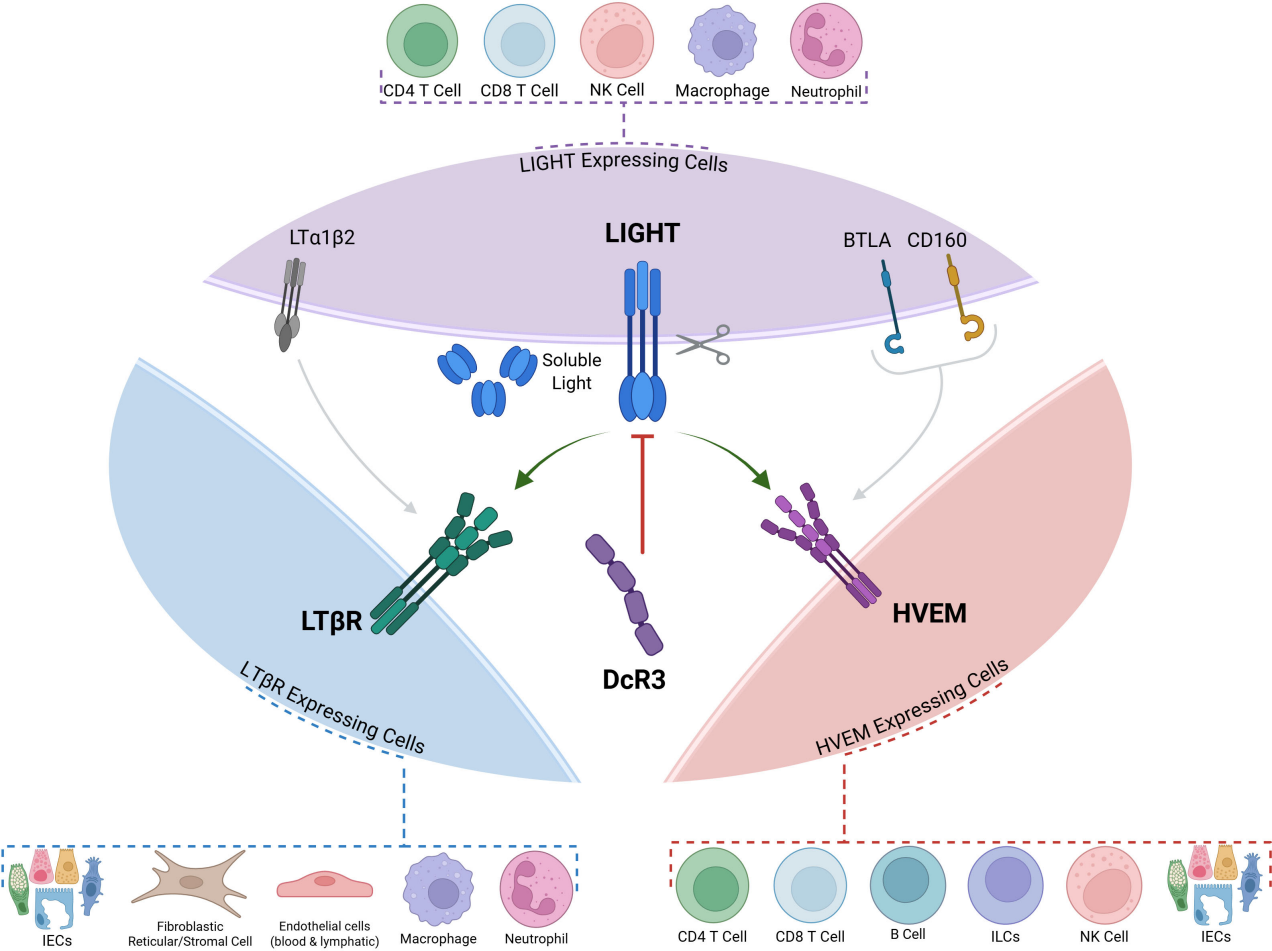
LIGHT (TNFSF14) and its Three Receptors: HVEM, LTBR and DcR3. LIGHT can interact with one of its three receptors: HVEM, LTBR and the decoy receptor DcR3. Membrane-bound LIGHT can be proteolytically cleaved to give rise to a soluble protein with distinct functions. Both BTLA and LTBR can engage with other ligands (BTLA and CD160 in the case of HVEM, and LTα1β2 in the case of LTBR). Dotted callouts summarize common cellular sources/targets: LIGHT-expressing cells—CD4^+^ T cells, CD8^+^ T cells, NK cells, macrophages, neutrophils; LTβR-expressing cells—intestinal epithelial cells (IECs), fibroblastic reticular/stromal cells, blood/lymphatic endothelial cells, macrophages, neutrophils; HVEM-expressing cells—CD4^+^ T cells, CD8^+^ T cells, B cells, ILCs, NK cells, IECs. LIGHT, lymphotoxin-like, inducible, competes with herpesvirus glycoprotein D for binding to HVEM; HVEM, herpesvirus entry mediator; LTβR, lymphotoxin-β receptor; DcR3, decoy receptor 3; BTLA, B and T lymphocyte attenuator; CD160, cluster of differentiation 160; ILCs, innate lymphoid cells; IECs, intestinal epithelial cells; LTα1β2, lymphotoxin α1β2

The tumour necrosis factor (TNF) superfamily, a diverse group of cytokines and receptors, is a key player in the human immune response, regulating immune cell activation, ontogeny and survival. Dysregulation of this family is often implicated in autoimmune and inflammatory conditions, making its members compelling therapeutic targets. Among these is the tumour necrosis factor superfamily member 14 (TNFSF14)—commonly known as LIGHT—a cytokine that is homologous to lymphotoxins and inducibly expressed during inflammation. The gene encoding LIGHT, *TNFSF14*, is located within a recognized IBD susceptibility locus on chromosome 19p13.3, providing a direct genetic link to the disease (5, 6). Like most TNF superfamily ligands, LIGHT is a type II transmembrane protein that can be proteolytically cleaved to release a soluble, active form (7) (**Figure 1**). In addition, alternative splicing of TNFSF14 can generate a distinct isoform lacking the transmembrane domain, which remains intracellular and may have unique regulatory functions (6).

LIGHT exerts its effects by signalling through two primary receptors: the Herpes Virus Entry Mediator (HVEM), broadly expressed on T cells and other immune cells, and the Lymphotoxin-β Receptor (LTβR), found predominantly on stromal and epithelial cells as well as on innate immune cells (8) (**Figure 1**). LIGHT can also bind to Decoy Receptor 3 (DcR3), a soluble protein encoded by the *TNFRSF6B* gene that acts as a decoy receptor for several TNF family ligands including, LIGHT, TL1A, and Fas ligand (9) (**Figure 1**).

Recent advances have revealed that LIGHT signalling is not limited to a simple dichotomy based on receptor engagement. Instead, both HVEM and LTβR pathways can mediate a range of immunological functions—from driving pro-inflammatory responses to promoting immune regulation, tissue repair, or fibrosis — depending on cellular context, ligand availability, and the local inflammatory milieu. For example, membrane−bound LIGHT binding to HVEM on effector T cells provides potent costimulatory signals and promotes T−cell activation and mucosal inflammation (10, 11), whereas HVEM expressed on regulatory T cells engages BTLA (B and T lymphocyte attenuator) on effector T cells to dampen their activation and reinforce immune tolerance (12). Similarly, LIGHT–LTβR signalling may facilitate epithelial regeneration and barrier repair (13, 14), yet also drives pathological tissue remodelling under chronic inflammatory conditions (15).

This context-dependent duality underscores the importance of LIGHT as a central regulator in intestinal immune homeostasis and pathogenesis. Adding another layer of complexity, recent single-cell RNA sequencing analyses of human ulcerative colitis tissue have identified a novel population of pro-inflammatory fibroblastic reticular cells that express both LIGHT and HVEM, suggesting a role for stromal-immune crosstalk in driving disease (16). A deeper understanding of these pathways is crucial for developing targeted therapies that can selectively modulate LIGHT’s effects, offering the potential for more precise and effective interventions in IBD.

## 2 Role of TNFSF14 (LIGHT) in intestinal inflammation and IBD

The intestinal immune system is a complex and highly regulated environment that must maintain a delicate balance between tolerance to beneficial commensal microbiota and robust immune responses against invading pathogens. In IBD, this critical balance is disrupted, leading to chronic and often debilitating inflammation.

**Figure 2:**
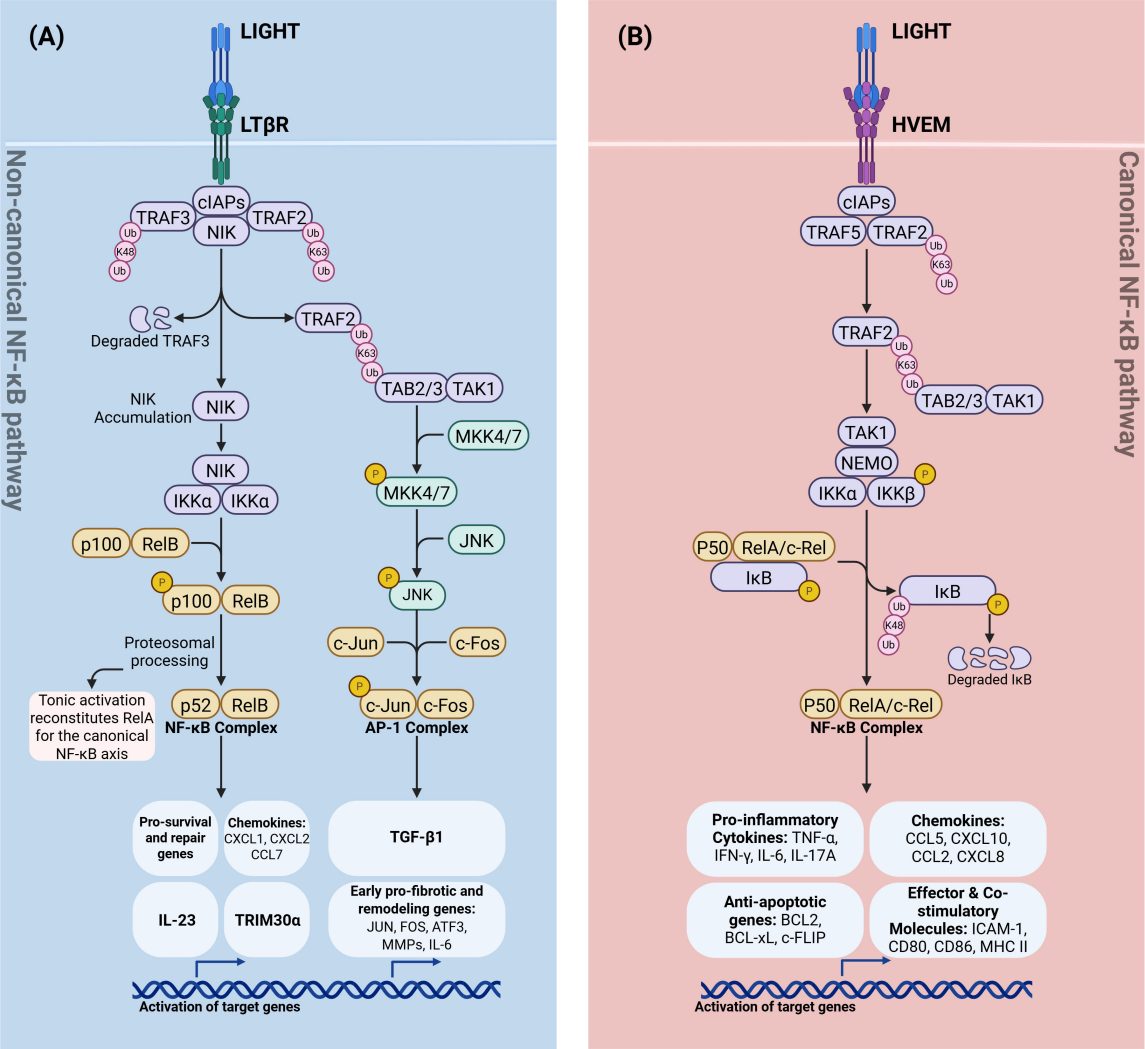
Divergent TNFSF14 (LIGHT) signaling through LTβR and HVEM. **(A)** LIGHT→LTβR (non-canonical NF-κB and AP-1 branch). LTβR ligation recruits TRAF2/3–cIAP1/2, leading to K48-linked ubiquitin–mediated degradation of TRAF3, NIK accumulation, and IKKα activation. IKKα drives p100 processing to p52 and RelB nuclear translocation (non-canonical NF-κB), inducing repair/homeostatic programs (e.g., IL-23, TRIM30α and select chemokines). In parallel, an LTβR–TRAF2–TAB2/3–TAK1 cascade activates MKK4/7→JNK, forming the AP-1 (c-Jun/c-Fos) complex and early pro-fibrotic/remodeling genes culminating in TGF-β1 transcription. Tonic non-canonical signaling replenishes latent RelA dimers available to the canonical NF-κB axis. **(B)** LIGHT→HVEM (canonical NF-κB branch). HVEM engages cIAPs and TRAF2/5 to signal via TAK1–NEMO–IKKα/β, phosphorylating IκB and targeting it for K48-linked degradation. Released p50–RelA/c-Rel dimers drive canonical NF-κB–dependent genes, including pro-inflammatory cytokines (TNF, IFN-γ, IL-6, IL-17A), chemokines (CCL5, CXCL10, CCL2, CXCL8), anti-apoptotic factors (BCL2, BCL-XL, c-FLIP), and effector/co-stimulatory molecules (ICAM-1, CD80, CD86, MHC II). Schematic is simplified; “P” indicates phosphorylation. K63-Ub marks signaling/scaffold ubiquitination; K48-Ub marks proteasomal degradation. LIGHT, lymphotoxin-like, inducible, competes with herpesvirus glycoprotein D for binding to HVEM; LTβR, lymphotoxin-β receptor; HVEM, herpesvirus entry mediator; NF-κB, nuclear factor-κB; NIK, NF-κB–inducing kinase; IKK, Inhibitor of κB kinase; NEMO, NF-κB essential modulator; AP-1, activator protein-1; TRAF, TNF receptor–associated factor; cIAP, cellular inhibitor of apoptosis protein; TAB, TAK1-binding protein; TAK1, transforming growth factor-β–activated kinase 1; MKK, mitogen-activated protein kinase kinase (MKK4/7); JNK, c-Jun N-terminal kinase; Ub, ubiquitin; K63-Ub, lysine-63–linked ubiquitin; K48-Ub, lysine-48–linked ubiquitin; p100/p52, NF-κB2 precursor/mature subunit; p105/p50, NF-κB1 precursor/mature subunit; RelA, NF-κB p65 subunit; RelB, NF-κB RelB subunit; c-Rel, NF-κB c-Rel subunit; IL-23, interleukin-23; TRIM30α, tripartite motif-containing 30α; TGF-β1, transforming growth factor-β1; TNF, tumor necrosis factor; IFN-γ, interferon-γ; IL-6, interleukin-6; IL-17A, interleukin-17A; CCL5, C-C motif chemokine ligand 5; CXCL10, C-X-C motif chemokine ligand 10; CCL2, C-C motif chemokine ligand 2; CXCL8, C-X-C motif chemokine ligand 8; BCL2, B-cell lymphoma 2; BCL-xL, B-cell lymphoma–extra large; c-FLIP, cellular FLICE-inhibitory protein; ICAM-1, intercellular adhesion molecule 1; CD80, cluster of differentiation 80; CD86, cluster of differentiation 86; MHC II, major histocompatibility complex class II.

Emerging evidence highlights the significant role of LIGHT signalling in initiating distinct downstream cascades highly relevant to the pathogenesis of IBD. On T cells, LIGHT-HVEM co-stimulation drives a potent Th1 inflammatory response characteristic of Crohn’s disease by enhancing the production of cytokines like IFN-γ and TNF-α (17–19) (**Figure 2B**). Within the intestinal epithelium, HVEM signalling shows further diversity that is independent of LIGHT; engagement of epithelial HVEM with CD160 on Intra-epithelial lymphocytes (IEL) activates a NIK-STAT3 axis for antimicrobial defense (20), while in intestinal progenitor cells it can promote tissue repair through activation of canonical NF-κB signalling (21).

Concurrently, activation of LTβR by its ligands predominantly triggers non−canonical RelB/p52 NF−κB signalling (**Figure 2A**). This pathway has several protective roles: in intestinal epithelial cells it promotes mucosal repair and proliferation after injury (22); it drives an IL−23/IL−22 circuit that enhances barrier regeneration and antimicrobial defence (23); and it induces neutrophil−attracting chemokines (CXCL1 and CXCL2) to support bacterial clearance and resolution of inflammation (24). LTβR signalling in macrophages also induces the NF−κB inhibitor TRIM30α, which dampens acute DSS−induced colitis (25).

Maladaptive effects occur when this signalling is persistent or excessive. Non−canonical Nfkb2 signalling can supplement latent NF−κB dimers and amplify canonical RelA responses, leading to heightened pro−inflammatory gene expression (26) (**Figure 2A**). This crosstalk between the canonical and non-canonical NF−κB pathways can prime epithelial and stromal cells for a hyper-inflammatory response. In chronic inflammation, high concentrations of LIGHT engage LTβR on macrophages, fibroblasts, and other structural cells, activating JNK/TGF−β1 axis that drives fibrotic responses (27). This results in excessive collagen deposition and extracellular matrix accumulation —hallmarks of tissue fibrosis (5, 15). Notably, blockade of either LIGHT or LTβR in experimental models reduces fibrosis and ameliorates disease severity (27, 28). The clinical relevance of these findings is underscored by the identification of both LTBR and NFKB2 as IBD risk loci (1). Thus, LIGHT–LTβR signalling orchestrates either tissue repair or pathology depending on the inflammatory context.

Immunohistochemical and histological studies in both human IBD and experimental colitis models have demonstrated that tissue localization and abundance of LIGHT, HVEM, and LTβR are dynamically altered during intestinal inflammation (**Figure 1**, **Table 1**). In colonic biopsies from patients with active Crohn’s disease and ulcerative colitis, immunostaining shows increased LIGHT expression on infiltrating T cells (38, 39), myeloid cells (13), and, less frequently, epithelial cells within inflamed mucosa (30). This staining is most intense in regions of active disease compared to adjacent non−inflamed areas (30, 37). Similarly, gene and protein analyses support increased LIGHT expression in IBD lesions, particularly within lamina propria leukocytes (34–37, 40). In murine models, these findings are robustly recapitulated: in DSS- and TNBS-induced colitis, LIGHT is markedly upregulated among CD45+ hematopoietic cells—including neutrophils and T cells—in the inflamed colon (29). Similarly, in the CD4+CD45RB^high^ T cell transfer model, LIGHT staining increases within inflammatory foci (13, 31). Transgenic overexpression of either murine or human LIGHT in murine T cells—driven by the *Lck* or *Cd2* promoter, respectively—leads to spontaneous intestinal inflammation with dense mononuclear infiltration and crypt damage (19, 41). Notably, the intensity and distribution of LIGHT and HVEM staining in these models typically parallels the severity of mucosal injury and leukocyte infiltration (13, 32, 37), with HVEM being detected on epithelial cells, fibroblasts, and immune infiltrates in both human and murine specimens (12, 36).

**Table 1:** Animal and Human Studies on LIGHT Signaling in IBD.

Study (Author, Year)	Study type	Model/Methods	Target(s) & Manipulation	Major outcome	Translational insight	Ref.
Shaikh et al., 2001	Animal	LIGHT transgenic mice (T cell- specific CD2 promoter)	Constitutive overexpression of human LIGHT on T cells.	Persistent T-cell LIGHT expression causes gut inflammation and lymphoid tissue destruction.	T cell–intrinsic LIGHT is sufficient to induce IBD-like pathology independent of external triggers.	(19)
Wang et al., 2005	Animal + Human	Adoptive T-cell transfer colitis; Crohn’s patient biopsies	Transfer of LIGHT-overexpressing CD4^+^ T cells into Rag1^-^/^-^ mice; assessed LIGHT expression in Crohn’s mucosa.	LIGHT-transgenic T cells induce colitis. No colitis occurs if host lacks LTβR or if donor T cells lack HVEM. Crohn’s patients show markedly elevated LIGHT in inflamed mucosa.	The LIGHT–HVEM/LTβR axis is central to Crohn’s-like IBD pathogenesis. Upregulated LIGHT in active Crohn’s suggests it as a biomarker and therapeutic target.	(17)
An et al., 2005	Animal	TNBS-induced colitis in rats	LTβR-Ig fusion protein (decoy receptor blocking LIGHT/LTαβ signaling) administered therapeutically.	Blocking LIGHT via LTβR-Ig reduces colitis severity. LTβR-Ig–treated rats showed milder colonic inflammation. Efficacy was comparable to standard therapy (mesalamine).	Validates LIGHT/HVEM as a therapeutic target in colitis: LTβR-Ig therapy ameliorated TNBS colitis.	(29)
Schwarz et al., 2007	Animal	Acute LIGHT challenge in mice; intestinal epithelial monolayers (Caco-2)	Exogenous LIGHT ± IFN-γ priming; used LTβR^-^/^-^ and HVEM^-^/^-^ mice; *in vitro* epithelial barrier assays.	LIGHT signals directly to intestinal epithelial LTβR to disrupt barrier function. No barrier loss occurs in LTβR^-^/^-^ mice, implicating LTβR as the critical receptor on epithelia.	Targeting MLCK or LIGHT–LTβR interactions may help preserve mucosal barrier function in IBD.	(30)
Steinberg et al., 2008	Animal	CD4^+^CD45RB^hi T-cell transfer colitis; DSS colitis in gene-knockout mice	HVEM-deficient T cells (donors) or HVEM^-^/^-^ hosts; also BTLA^-^/^-^ mice in DSS colitis.	Hosts lacking HVEM develop severe colitis. HVEM interaction with BTLA on T cells is required to prevent “runaway” intestinal inflammation. BTLA-deficient mice similarly fail to regulate colitis.	Identifies the HVEM–BTLA pathway as a key mucosal immune checkpoint. Enhancing BTLA–HVEM inhibitory signals may reinforce tolerance and prevent colitis flares.	(12)
Jungbeck et al., 2009	Animal	DSS-induced acute colitis in mice	Neutralized LIGHT with specific monoclonal antibodies; also utilized LIGHT^-^/^-^ mice	Neutralization or genetic ablation of LIGHT ameliorates DSS colitis. LIGHT^-^/^-^ mice showed dramatically less intestinal inflammation than WT. Anti-LIGHT mAb treatment in WT mice reduced disease severity.	Confirms LIGHT as a driver of intestinal inflammation. Highlights LIGHT as a promising therapeutic target for IBD.	(31)
Schaer et al., 2011	Animal	Two IBD models: (i) DSS colitis and (ii) CD45RB^hi T-cell transfer colitis in HVEM^-^/^-^ vs WT mice	HVEM^-^/^-^ mice lacking HVEM signaling in all cells	HVEM^-^/^-^ mice developed significantly milder DSS colitis and T-cell transfer colitis than WT.	Therapeutically, blocking HVEM–LIGHT co-stimulation might benefit IBD. Conversely, agonists of HVEM’s inhibitory ligand BTLA could mimic HVEM deficiency’s anti-inflammatory effect.	(32)
Krause et al., 2014	Animal	Chronic colitis models: CD4^+^CD45RB^hi T-cell transfer and DSS cycles using LIGHT-deficient vs WT mice	LIGHT^-^/^-^ in both T-cell transfer and DSS models; assessed LTβR vs HVEM roles	LIGHT^-^/^-^ mice developed more severe, non-resolving colitis. LIGHT signaling via LTβR on colon myeloid cells restrain innate immune cell activation and cytokine production, aiding recovery.	Uncovered a pro-resolving function of the LIGHT–LTβR pathway in mucosal immunity. Therapies must balance reducing pathogenic T-cell LIGHT signals while preserving its LTβR-mediated protective effects.	(13)
Riffelmacher et al., 2021	Animal	Conditional LTβR deletion in neutrophils; DSS colitis and mechanistic assays	Neutrophil-specific LTβR knockout; measured neutrophil metabolism ± LIGHT stimulation	Mice lacking LTβR in neutrophils developed more severe DSS colitis with massive neutrophil accumulation.	Therapies enhancing LIGHT–LTβR signals in neutrophils might help control colitis.	(33)
Fonseca Camarillo et al., 2020	Human	Gene expression profiling in colon biopsies from ulcerative colitis (UC) patients vs controls	Quantified mucosal mRNA levels of HVEM and other inflammatory mediators in patients vs healthy controls	HVEM gene expression is upregulated in ulcerative colitis colon mucosa (both active and quiescent) compared to controls.	HVEM may serve as a mucosal biomarker for UC. Aiding in stratification and treatment - identifying patients who might respond to HVEM/LIGHT blockage or BTLA modulating therapies.	(34)
Jaeger et al., 2021	Human	Single-cell RNA-seq + mass cytometry on ileal Crohn’s disease tissues (inflamed vs non-inflamed regions)	Profiled HVEM, BTLA, and CD160 expression and interactions at single-cell resolution; analyzed T-cell subsets and their receptor–ligand pairings.	Single-cell analysis revealed broad HVEM expression on intestinal epithelial and immune cells, with BTLA and CD160 on distinct T-cell subsets. Inflamed Crohn’s tissues showed altered T-cell subset distributions alongside changes in BTLA/HVEM ligand expression.	Modulating the HVEM–BTLA checkpoint could recalibrate T-cell responses in Crohn’s disease. HVEM-BTLA Checkpoint agonists or selective blockade of HVEM’s co-stimulatory ligand LIGHT might restore balance in the intestinal immune ecosystem.	(35)
Lushnikova et al., 2021	Human	Multiplex immunoassay of immune checkpoints in colon biopsies and sera	Measured HVEM and BTLA protein levels in samples from patients with UC and MC vs controls	BTLA levels were higher in active UC colon biopsies compared to controls and UC remission. HVEM levels, in contrast, did not rise in UC and tended to be lower in MC patients than in controls. In circulation, MC patients showed reduced BTLA and HVEM relative to healthy controls.	BTLA–HVEM checkpoint alterations correlate with disease type and activity: BTLA upregulation in active UC suggests an attempted compensatory inhibition or ongoing T-cell activation.	(36)
Cardinale et al., 2023	Human	Plasma LIGHT quantification in pediatric Crohn’s disease vs controls	Measured circulating *soluble LIGHT* levels by immunoassay; analyzed correlation with disease features	LIGHT is profoundly elevated in pediatric Crohn’s disease. LIGHT levels were high across disease subtypes and showed a positive correlation with blood neutrophil count. Even patients in clinical remission maintained higher LIGHT than controls.	Establishes LIGHT as a potential non-invasive biomarker and therapeutic target in Crohn’s disease. Measuring LIGHT levels may help track disease activity or identify patients who might respond to LIGHT-targeted therapy.	(37)

This table summarizes key preclinical and clinical studies demonstrating the dual pro-inflammatory and regulatory functions of LIGHT and its receptors, HVEM and LTβR. The models, key manipulations, major outcomes, and translational insights are presented.

BTLA, B and T lymphocyte attenuator; CD, Crohn’s disease; DSS, dextran sulfate sodium; HVEM, herpes virus entry mediator; IBD, inflammatory bowel disease; LTβR, lymphotoxin-β receptor; mAb, monoclonal antibody; MC, microscopic colitis; TNBS, trinitrobenzene sulfonic acid; UC, ulcerative colitis; WT, wild-type.

LTβR is mainly expressed by non-hematopoietic stromal and epithelial cells (13, 33, 40) (**Figure 1**). However, LTβR expression is also observed on mononuclear phagocytes and neutrophils, particularly within ulcerated or crypt-damaged regions in IBD patients and mouse models of chronic colitis, as demonstrated by immunohistochemical and protein-based analyses in both human and murine tissues (13, 33).

Studies in conditional knockout mice further highlight the protective role of LTβR on intestinal epithelium and neutrophils, with loss of LTβR leading to impaired mucosal healing, excess neutrophil accumulation, and aggravated tissue injury (13, 25, 33, 42). Overall, the consistent upregulation and context-specific localization of LIGHT, HVEM, and LTβR in sites of active intestinal inflammation across human disease and diverse colitis models underscores their central roles in orchestrating mucosal immune responses, leukocyte trafficking, and tissue injury in IBD.

### 2.1 HVEM pathway: pro-inflammatory and regulatory functions

HVEM (TNFRSF14) is a member of the tumour necrosis factor receptor superfamily and serves as a central hub and co-signalling molecular switch in mucosal immune regulation. HVEM is expressed broadly on T cells, innate lymphoid cells, and various other immune populations, and interacts with multiple ligands—including TNFSF14 (LIGHT), BTLA, and CD160—to deliver either co-stimulatory or inhibitory signals depending on the molecular context and cellular environment (12, 43–47) (**Figure 1**).

**Figure 3:**
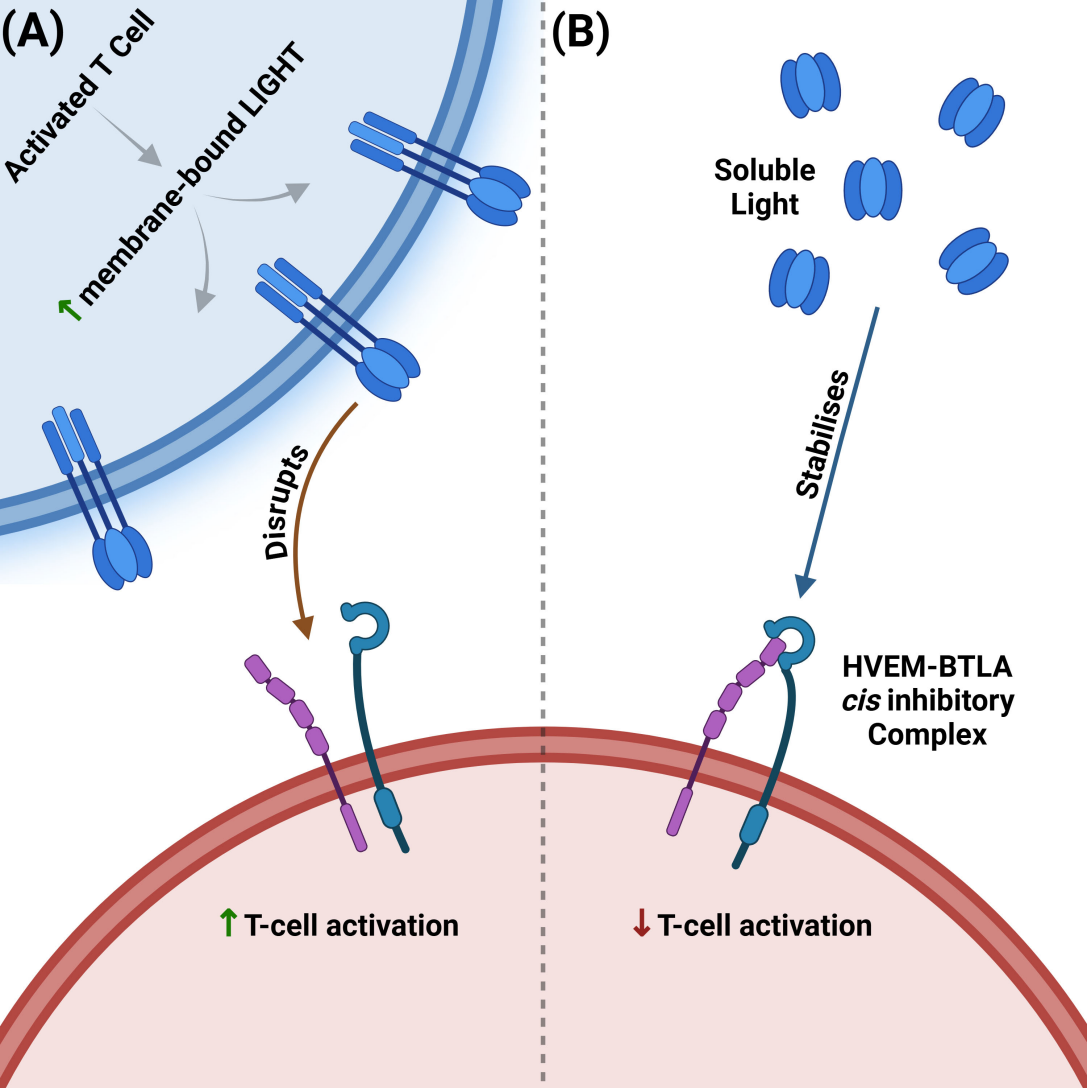
Opposing effects of membrane-bound vs soluble LIGHT on the HVEM–BTLA *cis* checkpoint. **(A)** Membrane-bound LIGHT disrupts the HVEM-BTLA inhibitory complex. Upon activation, T cells upregulate membrane-bound LIGHT, which disrupts the inhibitory HVEM–BTLA *cis*-complex. Disruption frees HVEM for trans-ligation by membrane-bound LIGHT, shifting signalling toward co-stimulation/activation. **(B)** Soluble LIGHT stabilizes the HVEM-BTLA inhibitory complex. Proteolytically shed soluble LIGHT stabilizes the inhibitory HVEM–BTLA *cis*-complex, which in turn blocks trans-ligation of membrane-bound LIGHT, maintaining the inhibitory signal. LIGHT, lymphotoxin-like, inducible, competes with herpesvirus glycoprotein D for binding to HVEM; HVEM, herpesvirus entry mediator; BTLA, B and T lymphocyte attenuator.

The LIGHT-HVEM axis is a potent driver of T-cell-mediated pathology in IBD (29). Upon activation, T-cells upregulate LIGHT expression, and engagement of membrane-bound LIGHT on activated T-cells with HVEM on naive CD4^+^ T cells sends a potent co−stimulatory signal that drives differentiation and expansion of Th1 and Th17 subsets and boost cytokine production (e.g., IFN−γ, TNF−α, IL-17); LIGHT–HVEM signalling also enhances proliferation and effector function of CD8^+^ cytotoxic T cells. Mechanistically, this activation occurs when membrane-bound LIGHT, transiently expressed on activated T cells, disrupts the inhibitory HVEM-BTLA complex on the same cell, displacing BTLA and thus lowering the threshold for T-cell activation (48) (**Figure 3**). This also allows effector T cells to overcome suppression by regulatory T cells (Tregs) (14, 17, 19). Indeed, LIGHT–HVEM interactions are critical for sustaining mucosal inflammation; in T-cell transfer models of colitis, HVEM-deficient T cells exhibit reduced proliferation and decreased expression of IL-6 and IL-23 receptors, which are essential for maintaining pathogenic Th17 cells (32). Conversely, LIGHT-deficient mice show impaired T-cell proliferation and cytokine secretion (49).

The severe consequences of dysregulated LIGHT signalling are highlighted in transgenic mouse models. In transgenic mice expressing the murine *Tnfsf14* gene under the control of a T−cell−specific *Lck* promoter, severe systemic autoimmune disease and intestinal inflammation develop spontaneously, characterised by a Th1−skewed cytokine profile and extensive immune−cell infiltration, as shown by *in vivo* phenotype analysis and histological examination (41). Bone marrow chimera experiments demonstrated that transferring bone marrow from LIGHT-transgenic donor mice (constitutively expressing LIGHT on T cells) into irradiated immunodeficient recipients was sufficient to induce inflammation, tissue destruction, and particularly severe intestinal inflammation in the recipients. These findings confirm that T-cell expression of LIGHT is sufficient to drive the disease phenotype, implicating LIGHT-expressing T- cells as the pathogenic driver of mucosal inflammation (19). It is noteworthy that these potent inflammatory effects in mouse models may be magnified by the absence of DcR3, that is naturally absent in mice (10).

Furthermore, adoptive transfer of naive T cells from LIGHT-transgenic mice into immunodeficient RAG-deficient recipients induces a rapid and severe intestinal inflammation that closely resembles Crohn’s disease, with hallmark features including transmural inflammation and a dominant Th1 immune response characterized by high levels of IFN-γ and TNF-α (17, 41).

Consistent with this, in the adoptive T-cell transfer model of colitis, naive CD4^+^CD45RB^high^ T cells from HVEM-deficient donor mice transferred into RAG-deficient recipients, are capable of inducing colitis with only a modest reduction in disease severity compared to wild-type donor T cells, whereas the absence of T−cell−derived LIGHT markedly blunts T−cell expansion and colonic inflammation (17). These findings indicate that LIGHT expression on T cells is essential for driving intestinal inflammation, while T-cell expression of HVEM provides additive co−stimulatory signals but is not required for disease (12). In addition to T cell responses, the LIGHT-HVEM network contributes to innate immune activation, amplifying inflammation through the secretion of LIGHT by effector cells like neutrophils and driving cytokine release from other immune cells, such as NK cells, which together fuel the pro-inflammatory environment typical of active IBD (10, 48).

HVEM signalling is also critical for the function of innate lymphoid cells (ILCs). The LIGHT-HVEM signalling axis, along with the inhibitory receptor BTLA, forms a key communication network that controls the activation state of ILCs, particularly RORγt+ ILC3s. In the context of host defence, this pathway drives the production of IFN-γ from ILC3s to protect against enteric bacterial infection (47, 50).

As previously mentioned, LIGHT–HVEM interactions in immune cells—most notably T lymphocytes—activates the canonical NF-κB signalling pathway (7, 51) (**Figure 2B**). Upon binding of LIGHT, HVEM recruits adaptor proteins such as TRAF2 and TRAF5, leading to downstream transcriptional programs that robustly induce pro-inflammatory cytokines, including interferon-gamma (IFN-γ) and tumour necrosis factor-alpha (TNF-α), as well as chemokines such as CCL5 and CXCL10 (51) (**Figure 2B**). This chemokine and cytokine milieu promotes the recruitment and activation of additional immune cells in the intestinal mucosa, amplifying and sustaining inflammatory responses. The ubiquitous expression of HVEM on lymphocytes ensures that LIGHT can efficiently drive adaptive immune activation, making this pathway a central contributor to the T cell–mediated pathology characteristic of IBD.

Despite its pro-inflammatory potential, the HVEM pathway is also critical for immune regulation and tolerance (12), primarily through its interaction with the inhibitory ligands BTLA and CD160 (43, 44, 46). The HVEM–BTLA axis functions as a critical immune checkpoint in the gut. In the adoptive T-cell transfer model of colitis, transfer of naive CD4^+^CD45RB^high^ T cells into HVEM-deficient recipient mice results in accelerated and lethal intestinal inflammation (12), underscoring a dominant protective role for HVEM expressed on host cells. This reflects HVEM’s role on recipient stromal and epithelial cells as an inhibitory ligand. In contrast to the earlier observation that HVEM−deficient donor T cells still induce colitis with only a modest reduction in severity—loss of HVEM on the host side removes this checkpoint entirely and precipitates uncontrolled inflammation (12, 32). This protection is mediated through engagement of BTLA on T cells, which suppresses excessive immune activation (51, 52). Indeed, it has been shown that when BTLA and HVEM are co-expressed on the same cell surface (“in *cis*”), they form a complex that strongly inhibits T-cell activation and restricts HVEM’s availability to bind membrane-bound LIGHT (43) (**Figure 3**). Intriguingly, this inhibitory state can be actively reinforced by soluble LIGHT, which, after being proteolytically cleaved from activated T cells, binds to and stabilizes the inhibitory HVEM-BTLA *cis*-complex without activating HVEM (**Figure 3**). This serves as a negative feedback mechanism to prevent excessive inflammation (48). Supporting this are studies using human T cell reporter systems and primary T cells showing that the HVEM-BTLA complex strongly inhibits T cell activation, even in the presence of exogenous soluble LIGHT, suggesting that this inhibition is not reversed by competing ligands (43) (**Figure 3**).

Among T cell subsets, HVEM-BTLA interactions are crucial for maintaining mucosal tolerance by both instructing the differentiation of peripheral Tregs and sustaining their suppressive function. Mechanistic studies in mice models show that engagement of HVEM on T cells by BTLA expressed on tolerogenic dendritic cells promotes the expression of *Foxp3*, a key step in the induction of extrathymic Tregs (44). In the adoptive T cell transfer colitis model, Tregs from HVEM-deficient mice showed impaired ability to suppress effector T cells, while BTLA-deficient effector T cells were resistant to Treg-mediated suppression when transferred into RAG-deficient hosts (12). These *in vivo* findings were corroborated by parallel *in vitro* co-culture suppression assays, which similarly demonstrated that Treg-mediated suppression depends on HVEM expression by Tregs and BTLA expression by effector T cells (53). Intriguingly, the stimulatory LIGHT-HVEM axis also plays a direct role in Treg biology, as LIGHT can act as a costimulatory molecule for Tregs (14), promoting their expansion and increasing Foxp3 expression (5). However, while LIGHT can co-stimulate Tregs and promote their expansion, its net effect is powerfully pro-inflammatory in environments where effector T cells are abundant, such as in inflamed tissue. In these settings, LIGHT provides such a potent stimulus that it allows effector T cells to overcome Treg suppression (14). It is plausible that this balance could shift depending on the local cellular composition. For instance, in healthy, non-inflamed tissue characterized by a high ratio of regulatory to effector T cells, the costimulatory action of LIGHT on the predominant Treg population might instead serve to reinforce local immune tolerance, resulting in a net regulatory outcome.

Clinical and genetic studies further support the role of HVEM as an immune checkpoint, with altered HVEM and BTLA expression profiles identified in patients with IBD and other gastrointestinal disorders (36, 54).

However, HVEM is not confined to immune cells; it is also expressed by intestinal epithelial cells (IEC), where it plays a critical role in innate mucosal defence. This pathway is independent of the canonical NF-κB and is critical for host defence against pathogenic bacteria like *Citrobacter rodentium*. Interaction of HVEM on IECs with CD160 on intraepithelial lymphocytes activates a NIK-STAT3 signalling axis, which in turn drives the expression of host defence genes against antimicrobial peptides (20, 45).

More recently, a second, distinct homeostatic function for epithelial HVEM has been described, which operates at steady state to promote the survival of intraepithelial T cells (IETs) through a LIGHT-dependent feedback loop. In this mechanism, LIGHT from mucosal lymphocytes engages HVEM on intestinal epithelial cells (IECs). This signal stimulates the IECs to synthesize and deposit basement membrane proteins, particularly collagen IV. This collagen then provides a crucial survival signal to IETs through its interaction with β1 integrins on their surface, revealing a novel mechanism by which LIGHT-HVEM signalling retains tissue-resident immune cells by modulating the structural microenvironment (55).

In summary, the HVEM pathway is a quintessential example of dual-function immune signalling in the gut. Its role is tightly controlled by the balance of available ligands, the responding cell type, and the local inflammatory environment. While LIGHT-HVEM engagement on effector lymphocytes robustly drives pro-inflammatory adaptive immune responses, the HVEM-BTLA axis acts as a dominant checkpoint to enforce tolerance and prevent excessive inflammation (43, 53). This functional complexity, further supported by altered checkpoint molecule expression in colitis patients (36), establishes HVEM as a central regulator of mucosal immunity and a complex but promising therapeutic target in IBD.

### 2.2 LTβR pathway: resolution of inflammation and fibrosis

The lymphotoxin β receptor (LTβR), is a key member of the TNF receptor superfamily encoded by the *LTBR/TNFRSF3* on chromosome 12, in a susceptibility locus associated with Crohn’s disease in large-scale human genetic studies (1). It is widely expressed on stromal and epithelial cells; among immune cells, its expression is particularly prominent on innate populations such as neutrophils, monocytes, and macrophages (33). LTβR interacts with two primary ligands: membrane-bound lymphotoxin α1β2 (LTα1β2) and LIGHT (TNFSF14) (24) (**Figure 1**), and the interplay between these two ligands is essential for the ontogeny of lymphoid structures. While LIGHT deficiency by itself does not impair lymphoid organ development, LIGHT and LTα1β2 have been shown to cooperate in the formation of mesenteric lymph nodes, demonstrating a partially redundant function *in vivo* (49). Both ligands can activate LTβR, but the context and cell type determine the outcome of this signalling, ranging from the resolution of inflammation (13, 22) to the promotion of tissue remodelling and fibrosis (26).

On epithelial cells, LTβR signalling is crucial in coordinating responses to infection. For instance, in a *Citrobacter rodentium* infection model, which serves as a surrogate model for human attaching-and-effacing pathogens like enteropathogenic and enterohemorrhagic E. coli (EPEC and EHEC) (56), protection is critically dependent on membrane LTα1β2 produced by RORγt+ innate lymphoid cells, while LIGHT is dispensable. LTα1β2 interacts with LTβR on intestinal epithelial cells to drive the production of the neutrophil-recruiting chemokines CXCL1 and CXCL2, which orchestrate the early innate immune response essential for bacterial clearance (24).

During intestinal injury and repair, the LTβR pathway is central to restoring mucosal integrity. Signalling through LTβR in intestinal epithelial cells (IECs) activates the non-canonical NF-κB pathway—particularly the RelB axis—which stimulates epithelial proliferation, crypt regeneration, and wound healing (**Figure 2A**). This protective signalling orchestrates the IL-23/IL-22 circuit, whereby LTβR-driven IL-23 production by IECs stimulates group 3 innate lymphoid cells (ILC3s) to secrete IL-22, a critical cytokine for barrier regeneration (42, 57). While both ligands are upregulated after injury, LIGHT appears to be a key driver of this repair process. This is demonstrated in both DSS-induced and MTX-induced mice models, where absence of LIGHT results in persistent, unremitting intestinal inflammation. This is marked by increased infiltration of neutrophils and monocytes and elevated chemokine expression (CXCL1, CXCL2, CCL7), highlighting a critical role for LIGHT–LTβR signalling in resolving gut inflammation (13, 22). Collectively, these findings indicate that LTα1β2–LTβR signalling in intestinal epithelial cells is the principal driver of the early inflammatory response required for pathogen clearance, whereas LIGHT–LTβR signalling, while dispensable for the initiation of inflammation, is essential for resolving gut inflammation and restoring mucosal homeostasis during tissue repair.

Conversely, dysregulated or chronic LTβR signalling can be pathogenic, as it can prime IECs for a hyper-inflammatory response through crosstalk between NF-κB pathways. As previously mentioned, tonic activation of the non-canonical pathway supplements the pool of latent canonical RelA dimers, leading to exacerbated pro-inflammatory gene expression and worsened colitis upon a subsequent inflammatory trigger (26) (**Figure 2A**). The clinical relevance of this is underscored by the identification of IBD-associated variants in/proximal to both LTBR and NFKB2 (1).

Under conditions of chronic inflammation, LIGHT-LTβR signalling also drives pathological tissue remodelling and fibrosis. By activating the JNK/TGF-β1 axis in fibroblasts and other structural cells, this pathway promotes excessive collagen deposition and the accumulation of extracellular matrix, contributing to fibrogenesis (5, 15) (**Figure 2A**). Blockade of either LIGHT or LTβR in these models reduces fibrosis and ameliorates disease severity (27, 28). Furthermore, in IECs, LIGHT signalling via LTβR in the presence of IFN-γ can synergistically disrupt the cytoskeleton and increase mucosal permeability, directly compromising barrier function (30).

In addition to its functions on epithelial and stromal cells, LTβR signalling on innate immune cells can also contribute to the resolution of inflammation. For example, in neutrophils, LTβR activation is protective, modulating metabolism to suppress reactive oxygen species (ROS) and thereby limiting oxidative tissue damage (10, 33). Signalling in macrophages is also protective; LTβR activation on macrophages has been shown to ameliorate acute colitis by inducing the expression of TRIM30α, a negative regulator that inhibits NF-κB activation and subsequent pro-inflammatory cytokine production (25). These cell-type-specific and context-dependent outcomes highlight the functional plasticity of the LTβR pathway (5, 30).

The complex nature of LTβR signalling—facilitating tissue repair and barrier restoration, while potentially driving fibrotic responses — reflects the fact that these two processes are intimately intertwined; as the acute inflammatory response subsides, healing processes kick in. However, with disease progression, the fibrotic tissue becomes of itself a driver for some of the complications seen in patients (for example, malabsorption). The overall outcome is dictated by the balance of ligand expression, receptor availability, inflammatory milieu, and disease chronicity (22, 27, 33). This spatio-temporal context dependence makes LTβR both a promising and a challenging target for therapeutic modulation.

### 2.3 Understanding LIGHT signalling in IBD

The LIGHT (TNFSF14) signalling axis illustrates how immune responses in the gut are shaped by both cellular and environmental factors. Through HVEM and LTβR, LIGHT can either drive inflammation or promote tissue repair and immune tolerance (**Figure 2**), depending on the particular receptor involved and the disease stage present within the intestinal microenvironment (12, 22, 33, 45, 58).

Pro-inflammatory functions of LIGHT are primarily mediated by its interaction with HVEM on T cells and innate immune cells. This engagement promotes Th1 and Th17 differentiation, enhances effector cytokine production, and sustains chronic inflammation typical of active IBD. Conversely, the HVEM-BTLA axis serves as a checkpoint that can limit immune activation and promote tolerance, particularly in steady-state or regulatory settings (43, 53, 58). Recent mechanistic data reveal that BTLA’s inhibitory signalling through HVEM is dominant even in the presence of costimulatory ligands, safeguarding against unchecked inflammation and highlighting the importance of molecular context in determining HVEM function (43). The functional separation of these pathways has been elegantly demonstrated using knock-in mice with HVEM mutants that can bind either LIGHT or the inhibitory ligands BTLA/CD160, but not both. In these models, the specific inflammatory context dictates which HVEM interaction is critical. For example, host defence against *Yersinia enterocolitica* infection required the pro-inflammatory LIGHT-HVEM interaction to drive IFN-γ from ILC3s (47), while in contrast, the suppression of T-cell mediated autoimmune hepatitis relied exclusively on the inhibitory HVEM-BTLA/CD160 checkpoint (46).

This highlights an additional layer of complexity, as the nature of the pathogen determines which HVEM interaction predominates in mucosal defence; protection against *Yersinia enterocolitica* requires HVEM-LIGHT signalling, whereas protection against *Citrobacter rodentium* requires the HVEM-CD160 pathway (20, 46). The complexity extends to the interplay between LIGHT and its related ligand, LTα1β2, at their shared receptor, LTβR. The ultimate outcome of LTβR signalling is not determined by a single ligand, but rather by the balance and stoichiometry of both. This is highlighted by the paradoxical finding in some colitis models that, while deleting either LIGHT or LTα1β2 individually worsens disease, the simultaneous deletion of both ligands is protective. This suggests that the relative abundance of different ligands can fundamentally alter the signalling output of the receptor (38).

As previously detailed, LIGHT–LTβR signalling in intestinal epithelial cells is essential for mucosal repair and barrier restoration in models of injury. However, when this pathway is chronically or aberrantly activated, it can also drive tissue remodelling and fibrosis through profibrotic mechanisms, including the JNK/TGF-β1 axis (22, 27, 28, 33). The necessity of both receptor pathways to drive pathology is powerfully illustrated in the LIGHT-transgenic T-cell transfer model of colitis (17). In this model, severe disease requires LIGHT to engage two distinct cell types through its two different receptors. Colitis was significantly ameliorated when the donor T cells lacked HVEM, demonstrating the need for T-cell co-stimulation. However, the disease was completely abrogated when the recipient mice lacked LTβR, proving that LIGHT signalling onto non-T cells (likely stromal or epithelial cells) is also essential for the full development of intestinal inflammation. This finding confirms that the maximum pathogenic effect of LIGHT requires the integration of signals through both the HVEM and LTβR pathways (17).

As previously discussed, LIGHT signalling promotes tissue protection and repair during resolution but, when sustained, can instead drive pathological tissue remodelling and complications (26). This functional complexity is supported by clinical findings showing altered expression of HVEM, BTLA, and LIGHT in patients with IBD and related gastrointestinal disorders at different disease stages (36, 53, 54). In one example, studies in patients with microscopic colitis show altered immune checkpoint profiles, including varied expression levels of HVEM and BTLA in colon biopsies (36).

Therapeutic targeting of the LIGHT axis in IBD thus requires a nuanced understanding of its dual functions. Selective modulation of LIGHT-HVEM or LIGHT-LTβR signalling—enhancing regulatory/tolerogenic outcomes while dampening pro-inflammatory or fibrotic responses—represents a promising but complex strategy for restoring mucosal homeostasis and preventing chronic intestinal damage (22, 27, 33, 58).

## 3 Therapeutic implications and clinical studies

Despite recent advances, there remains an unmet need for novel therapies in IBD that can be used in patients who fail to respond to currently approved drugs (including TNFα inhibitors) (41). Many of these patients are primary non-responders whose inflammation is likely driven by non-TNF-dependent pathways, highlighting the need for therapies with alternative mechanisms of action (59).

The complex role of LIGHT in immune regulation and inflammation makes it an attractive target for therapeutic intervention in IBD. However, given its dual capacity to either promote or resolve inflammation, targeting this pathway can be a double-edged sword, and therapeutic strategies must be carefully designed to selectively modulate its downstream signalling pathways. So far, the focus has largely been on inhibiting LIGHT to mitigate its pro-inflammatory effects, particularly those mediated through HVEM (5, 18).

The rationale behind these efforts is the idea that blocking LIGHT would serve to control T cell activation and proliferation, especially in conditions where the LIGHT-HVEM axis is overactive (19). This strategy aims to reduce the pro-inflammatory signals that contribute to chronic inflammation in IBD, and is supported by preclinical data demonstrating that therapeutic blockade of LIGHT with neutralizing monoclonal antibodies ameliorates experimental colitis in acute DSS models (29, 31).

Building on the promising preclinical data and the pressing need for alternative therapies in patients who do not respond to anti-TNFα treatment (**Table 1**), novel agents such as CERC-002, an investigational fully human anti-LIGHT (TNFSF14) monoclonal antibody, have been developed. This first-in-class agent offers a novel mechanism of action for patients with biologic-refractory disease by reducing circulating LIGHT levels and mitigating its pro-inflammatory effects. Promising results emerged from a phase 1b trial of CERC-002 in patients with moderate-to-severe Crohn’s disease who had previously failed anti-TNFα therapy. In this small cohort, treatment reduced circulating LIGHT levels, demonstrating target engagement. Furthermore, 75% of participants showed clinically meaningful endoscopic improvement, and the therapy had a favourable safety profile with no significant adverse events (59). Despite these encouraging findings in a biologic-refractory population, the trial (ClinicalTrials.gov identifier: NCT03169894) was terminated early by the sponsor due to a strategic pipeline re-prioritization (i.e. a business decision to shift focus).

This same antibody (also known as AVTX-002) has since shown a significant protective effect in a randomized phase II trial for hospitalized patients with COVID-19-associated pneumonia and ARDS (60). Both LIGHT receptors, HVEM and LTβR, are broadly expressed in lung tissues (on infiltrating myeloid cells and alveolar epithelial cells), and LIGHT has long been studied in the context of pulmonary fibrosis (60, 61). Engagement of these receptors by LIGHT triggers NF-κB–dependent inflammatory cascades, driving release of cytokines/chemokines and recruitment of neutrophils and T cells that damage the lung epithelium (60). This is supported by the observation that patients with COVID-19-associated pneumonia and ARDS have markedly elevated free LIGHT levels in serum. These findings provide strong clinical evidence for the pathogenic role of LIGHT in acute, severe inflammatory conditions at barrier organs (10).

Despite these promising preliminary results, any targeting of this this axis must be carefully examined given the increased risk of colorectal cancer (CRC) in patients with IBD (62).

Within the tumour microenvironment, BTLA-HVEM signalling plays a critical role in suppressing anti-tumour immunity: HVEM expressed by tumour or stromal cells engages BTLA on T cells, driving T cell exhaustion (63–65). As a result, therapeutic interventions that indirectly enhance BTLA-HVEM inhibitory signalling—such as LIGHT blockade—may further diminish anti-tumour immune surveillance in an already at-risk population.

Adding to this complexity, large-scale analyses of CRC patient data have identified high expression of LTβR as an independent risk factor associated with worse overall survival (66). It is unclear how global LIGHT blockade would impact the bioavailability of other ligands (mainly LTα1β2) engaging with the LTβR receptor at the tumour site. Therefore, careful risk assessment and long-term monitoring are warranted when considering anti-LIGHT therapies in IBD patients.

Beyond global LIGHT blockade, future therapeutic strategies will likely focus on more precise interventions (67). A promising avenue is selective receptor modulation, leveraging structural biology insights that reveal distinct, non-overlapping binding sites on HVEM for its pro-inflammatory (LIGHT) versus inhibitory (BTLA) ligands (46). This provides a clear molecular rationale for developing novel therapeutic modalities—including peptide inhibitors and small molecules—that can specifically block T-cell activation via the LIGHT-HVEM interface while preserving crucial immune checkpoints (46, 68). An alternative and more established approach involves targeting key intracellular signalling effectors downstream of the LIGHT receptors. This can be achieved by developing small molecules that disrupt the protein-protein interactions (PPIs) of receptor-proximal adaptors, such as TRAF2, TRAF3, and TRAF5, which are directly recruited to HVEM and LTβR (51, 69, 70). While developing inhibitors for these specific PPIs is challenging, the successful development of small-molecule inhibitors against related family members, such as TRAF6, provides a strong proof-of-concept that this target class is amenable to therapeutic modulation by small molecules (71). Furthermore, small molecule inhibitors can be used to counteract the specific pathological consequences of dysregulated LIGHT signalling. For example, under conditions of chronic inflammation, persistent LIGHT-LTβR signalling can drive fibrosis via the JNK pathway (27) or amplify pro-inflammatory canonical signalling through non-canonical NF-κB crosstalk, a mechanism shown to exacerbate experimental colitis (26). Potent small molecule inhibitors of these downstream kinases are in development, and their therapeutic potential in IBD is underscored by both pharmacological and genetic studies. For instance, the JNK-inhibiting peptide, D-JNKI-1, clinically attenuates chronic DSS-induced colitis, and genetic deletion of JNK2 strongly mitigates TNF-driven Crohn’s-like ileitis in mice (72, 73). Likewise, inhibitors of the upstream kinase NIK have proven effective in preclinical models of lupus and liver inflammation (74, 75).

Finally, a crucial future direction will be combination therapy, particularly for the biologic-refractory population (67). Integrating a LIGHT pathway modulator with an existing treatment offers a multi-pronged approach to overcome therapeutic resistance. For instance, combining anti-LIGHT therapy with an anti-integrin agent would simultaneously target T-cell co-stimulation and gut-specific leukocyte homing. Alternatively, a combination with a JAK inhibitor would provide a comprehensive vertical blockade, targeting an upstream signal (LIGHT) and the downstream intracellular pathways used by numerous other cytokines (10, 76).

## 4 Conclusion

TNFSF14 (LIGHT) is increasingly recognized as a multifaceted regulator in IBD, capable of amplifying inflammation via HVEM on effector T cells, while also facilitating immune tolerance and resolution through LTβR and, in some settings, HVEM on regulatory T cells (**Table 1**). While clinical data suggests that LIGHT is upregulated in IBD and that it correlates with inflammation, preclinical models reveal a more complex picture, with both pathogenic and protective roles that vary depending on disease stage and cellular context.

This duality presents a central challenge for therapeutic targeting. While early clinical trials of anti-LIGHT antibodies support its potential as a therapeutic axis, a strategy of global LIGHT blockade risks disrupting essential homeostatic functions like tissue repair and possibly anti-tumour surveillance. The future of LIGHT-targeted therapy in IBD must therefore focus on distinguishing its detrimental and beneficial effects — particularly pathogenic HVEM signalling versus pro-resolving LTβR pathways.

Ultimately, LIGHT exemplifies the complexity of immune regulation in IBD, and successful clinical translation will require not only innovative drug design but also a nuanced understanding of the contextual signals that shape LIGHT’s activity, enabling the development of strategies that selectively modulate the pathway to restore immune homeostasis without compromising its protective functions.
